# The Structural Abnormalities Are Deeply Involved in the Cause of *RPGRIP1*-Related Retinal Dystrophy in Japanese Patients

**DOI:** 10.3390/ijms241813678

**Published:** 2023-09-05

**Authors:** Kaoruko Torii, Sachiko Nishina, Hazuki Morikawa, Kei Mizobuchi, Masakazu Takayama, Nobutaka Tachibana, Kentaro Kurata, Akiko Hikoya, Miho Sato, Tadashi Nakano, Maki Fukami, Noriyuki Azuma, Takaaki Hayashi, Hirotomo Saitsu, Yoshihiro Hotta

**Affiliations:** 1 Department of Ophthalmology, Hamamatsu University School of Medicine, 1-20-1 Handayama, Higashi-ku, Hamamatsu 431-3192, Japan; 2 Division of Ophthalmology, National Center for Child Health and Development, 2-10-1 Okura, Setagaya-ku, Tokyo 157-8535, Japan; 3 Department of Ophthalmology, The Jikei University School of Medicine, 3-25-8 Nishi-shimbashi, Minato-ku, Tokyo 105-8461, Japan; 4 Department of Molecular Endocrinology, National Research Institute for Child Health and Development, 2-10-1 Okura, Setagaya-ku, Tokyo 157-8535, Japan; 5 Department of Developmental and Regenerative Biology, Medical Research Institute, Tokyo Medical and Dental University, 1-5-45 Yushima, Bunkyo-ku, Tokyo 113-8510, Japan; 6 Department of Biochemistry, Hamamatsu University School of Medicine, 1-20-1 Handayama, Higashi-ku, Hamamatsu 431-3192, Japan

**Keywords:** Leber congenital amaurosis (LCA), *RPGRIP1*, whole-exome sequencing (WES), whole-genome sequencing (WGS), structural abnormalities, prevalent variant, retinal layer structure

## Abstract

Leber congenital amaurosis (LCA) is the most severe form of inherited retinal dystrophy. *RPGRIP1*-related LCA accounts for 5–6% of LCA. We performed whole-exome sequencing and whole-genome sequencing (WGS) on 29 patients with clinically suspected LCA and examined ophthalmic findings in patients with biallelic pathogenic variants of *RPGRIP1*. In addition to five previously reported cases, we identified five cases from four families with compound heterozygous *RPGRIP1* variants using WGS. Five patients had null variants comprising frameshift variants, an *Alu* insertion, and microdeletions. A previously reported 1339 bp deletion involving exon 18 was found in four cases, and the deletion was relatively prevalent in the Japanese population (allele frequency: 0.002). Microdeletions involving exon 1 were detected in four cases. In patients with *RPGRIP1* variants, visual acuity remained low, ranging from light perception to 0.2, and showed no correlation with age. In optical coherence tomography images, the ellipsoid zone (EZ) length decreased with age in all but one case of unimpaired EZ. The retinal structure was relatively preserved in all cases; however, there were cases with great differences in visual function compared to their siblings and a 56-year-old patient who still had a faint EZ line. Structural abnormalities may be important genetic causes of *RPGRIP1*-related retinal dystrophy in Japanese patients, and WGS was useful for detecting them.

## 1. Introduction

Leber congenital amaurosis (LCA; MIM#204000) is the most severe form of inherited retinal dystrophy (IRD) and is characterized by nystagmus, a lack of pursuit, and a severely reduced or absent electroretinogram (ERG) from birth, accompanied by severe visual impairment [1,2]. Autosomal dominant forms have been reported; however, most are autosomal recessive, and 25 causative genes have been reported [3,4,5]. *RPGRIP1*-related LCA (LCA6; #613826) reportedly accounts for 5–6% of LCA [3,4,5,6]. *RPGRIP1* variants have been reported to cause IRD, including retinitis pigmentosa (RP; #268000), cone–rod dystrophy (CRD; #120970), LCA, and pigmented paravenous retinochoroidal atrophy (PPRCA), and genetic and clinical data have been accumulated [7,8,9,10,11,12]. *RPGRIP1* plays a key role in connecting the cilia of photoreceptors, and *RPGRIP1* deficiency makes it difficult to maintain light-sensitive outer segments [13,14]. In LCA6, although the retinal function is lost during childhood, the retinal structure is relatively retained until adulthood [8]. These characteristics are advantageous for gene therapy.

The yield of genetic diagnoses has improved owing to the increased availability of whole-genome sequencing (WGS) and whole-exome sequencing (WES), and also due to the reduction in cost and the rapid progress of various data analysis technologies. WGS can detect structural abnormalities, transposon insertions, and deep-intronic splicing variants that are impossible or difficult to detect using WES. In cases where causative variants cannot be detected via WES, the chance of detecting them increases when performing WGS [9,15,16,17,18]. We believe WGS is effective for cases of suspected autosomal recessive disorders in which a likely pathogenic variant is found in one allele with no abnormality detected in another allele. Furthermore, the risk assessment system based on genetic counseling has improved due to enhancing databases of healthy individuals.

Previously, we examined five individuals from three families with biallelic *RPGRIP1* variants and found that retinal function was severely impaired in childhood, whereas the retinal structure was relatively preserved [19,20]. By performing WES/WGS on clinically suspected LCA cases without systemic diseases other than psychomotor retardation, we identified LCA caused by *RPGRIP1* variants in five cases from four families. In particular, a 1339 bp deletion encompassing exon 18 of *RPGRIP1* was observed in three individuals from two families. This deletion is relatively prevalent in the Japanese population (allele frequency: 0.002) and possibly in Japanese patients with *RPGRIP1*-related retinal dystrophy. Furthermore, microdeletions involving exon 1 were observed in four cases. Because LCA is a rare disease, the number of subjects is too small to draw conclusions; nonetheless, structural abnormalities may be a major cause in *RPGRIP1*-related retinal dystrophy in the Japanese population. We also analyzed detailed clinical findings during the follow-up period in 10 patients, one of whom was 56 years old. This report provides important information for both genetic counseling and gene therapy.

## 2. Results

### 2.1. Pathogenic Variants in the Subjects

**Figure 1:**
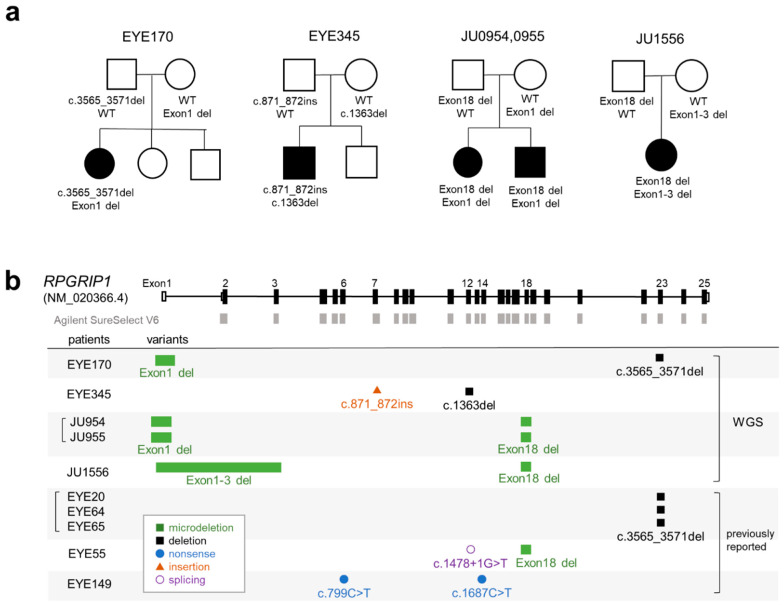
Pedigrees and schematic representation of *RPGRIP1* variants. (**a**) Pedigree of five patients with biallelic *RPGRIP1* variants. The segregation of each variant is shown. (**b**) Schematic representation of *RPGRIP1* transcript (NM_020366.4). The UTR and coding region are open and filled rectangles, respectively. Baits of Agilent SureSelect V6 are shown in gray boxes. *RPGRIP1* variants in five patients diagnosed using WGS are shown together with variants in five patients from our previous report.

In this study, we performed WES on 29 patients with clinically suspected LCA without systemic symptoms other than psychomotor delay (see Section 4 for details). WES identified two patients with heterozygous frameshift variants in one *RPGRIP1* allele (EYE170 and 345); however, no abnormalities were found in the other allele. Therefore, we performed WGS on 14 unsolved cases, including EYE170 and 345. Table 1 shows a summary of the patients with pathogenic variants of *RPGRIP1* (see also Supplementary Table S1). We identified five cases from four families with biallelic pathogenic variants (Figure 1a). EYE170 had a heterozygous microdeletion involving exon 1 of *RPGRIP1* (NM_020366.4), detected using two tools, Canvas [21] and Manta [22]. Exon 1 of NM_020366.4 is a newly annotated non-coding exon that was absent in the previous version (NM_020366.3) and was not covered by the SureSelect Human All Exon V6 kit (Figure 1b). EYE345 had a heterozygous *Alu* insertion that was detected using TEMP2 [23]. The remaining three cases (JU0954, 0955, and 1556) from two families had compound heterozygous microdeletions involving one to three exons (Figure 1b). Notably, a 1339 bp deletion involving exon 18 was identified in three cases via Manta. We previously reported five cases from three families with biallelic *RPGRIP1* variants [19,20] (Figure 1b), including one case with a deletion involving exon 18, suggesting that microdeletion may be a major genetic cause of LCA in Japanese patients.

**Figure 2:**
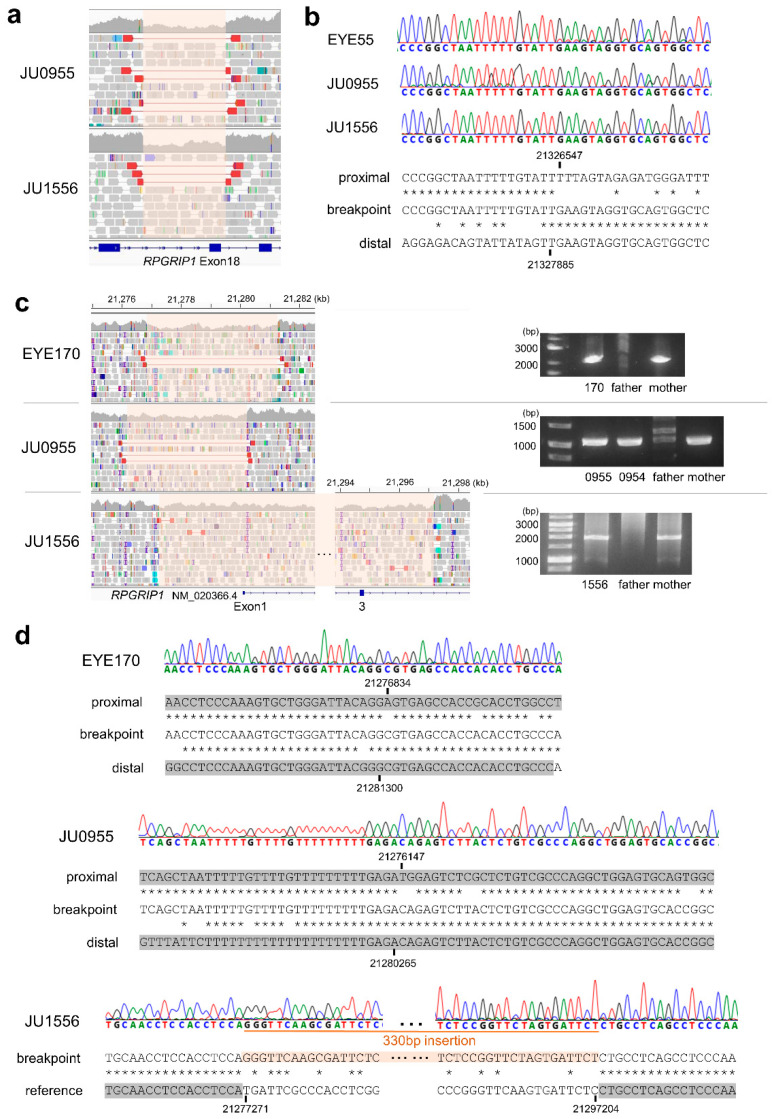
Microdeletions involving *RPGRIP1* exons. (**a**) The IGV view of a microdeletion involving exon 18. The deletion interval is highlighted in pink. (**b**) Breakpoint sequences in three patients showing an identical 1399 bp deletion. An asterisk indicates a sequence match between the upper and lower nucleotides. (**c**) The IGV view of microdeletions involving exon 1 (**left**) and results of breakpoint PCR (**right**). (**d**) Breakpoint sequences in three patients suggesting *Alu*-mediated recombination in three cases. *Alu* sequences are highlighted in gray. An asterisk indicates a sequence match between the upper and lower nucleotides.

**Figure 3:**
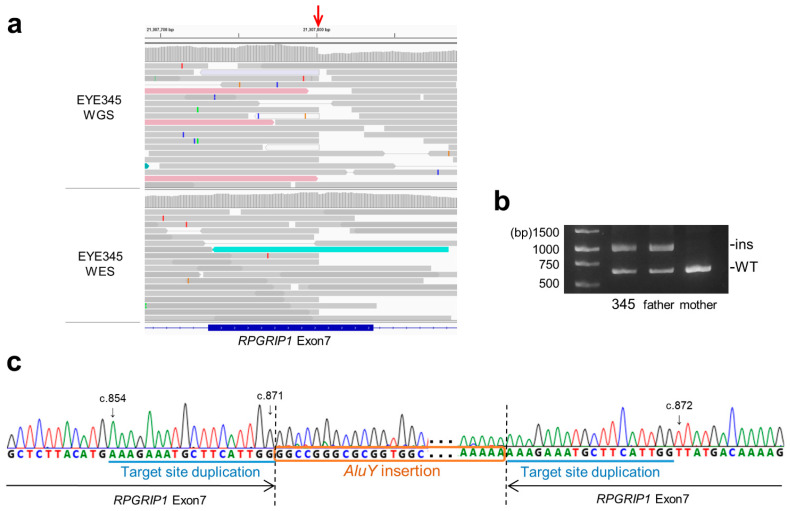
*Alu* insertion in *RPGRIP1* exon 7. (**a**) The IGV view shows an abrupt decrease (red arrow) in the depth of *RPGRIP1* exon 7 in WGS data. However, such a decrease is subtle in WES data, suggesting that inserted DNA fragments were not efficiently captured. (**b**) Breakpoint PCR shows paternally transmitted insertion that is approximately 400 bp in size. (**c**) Breakpoint sequences show *AluY* insertion with 18 bp target site duplication.

Next, we characterized the breakpoint sequences by de novo assembly using aberrantly aligned read pairs identified by manual inspection using an integrative genomics viewer (IGV), and confirmed these using breakpoint-specific polymerase chain reaction (PCR) (Supplementary Table S2). The microdeletion involving exon 18 was 1339 bp in size (Figure 2a; NC_000014.9: g.21326547_21327885del). Using breakpoint PCR and Sanger sequencing, we confirmed that the microdeletions were identical among four patients (EYE55, JU0954, 0955, and 1556) (Figure 2b). Microdeletions were not observed in gnomAD structural variants (SVs) v2.1; however, they were found in 1 of 444 alleles in JSV1 (long-read sequencing of Japanese, ID: JSV1_hg38_DEL.10214) and 33 of 16,760 alleles in 8.3KJPN-SV (short-read sequencing of Japanese, ID:111853). Therefore, microdeletions involving exon 18 are considered relatively prevalent in the Japanese population (allele frequency = 0.002). Breakpoints of a 4466 bp deletion (EYE170; NC_000014.9: g.21276834_21281300del) and a 4119 bp deletion (JU0954 and 0955; NC_000014.9: g.21276147_21280265del), both involving exon 1, were localized in the *Alu* sequence (Figure 2c). Breakpoints of a 19,934 bp deletion involving exons 1–3 (NC_000014.9: g.21277271_21297204delinsN [330]) were also localized in the *Alu* sequence (Figure 2d), suggesting that *Alu*-mediated recombination might be frequent in the vicinity of exon 1. Consistent with this, a 4421 bp deletion (chr14:21276854-21281275, ID:111850) was found in three of 16,760 alleles in 8.3KJPN-SV, suggesting that microdeletions involving exon 1 should be carefully examined. In EYE345, an *Alu* insertion was detected by TEMP2; the IGV showed an abrupt decrease in the depth of *RPGRIP1* exon 7, which was unclear from the WES data (Figure 3a). De novo assembly and breakpoint PCR confirmed an approximate 400 bp *AluY* insertion with an 18 bp target site duplication (Figure 3b,c), suggesting that transposition occurred via LINE-1. In summary, structural abnormalities were found in six cases from five families, including microdeletions involving exon 1 or exon 18 and an *Alu* insertion.

### 2.2. Clinical Findings of Participants

Ten patients with biallelic pathogenic variants of *RPGRIP1* ranged in age from 3 months to 50 years at their first visit and from 10 to 56 years at their last visit, with a follow-up period ranging from 6 to 20 years. Ophthalmological measurements of the patients, including best-collected visual acuity (BCVA), visual field, and electrophysiological findings, are summarized in Table 2.

**Figure 4:**
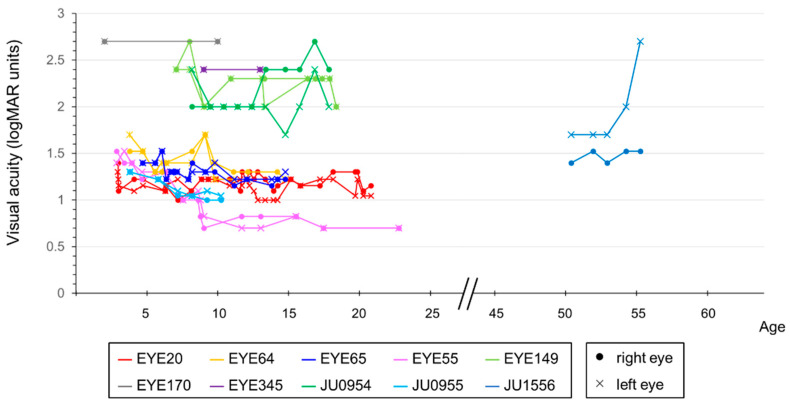
Correlation between visual acuity and age in patients with *RPGRIP1* variants. The graph compares the visual acuity values, in logMAR units, with age. Lines indicate data from the corresponding person and eye. Different colors are used to represent each person, with dots indicating data for the right eye and crosses indicating data for the left eye. Although visual acuity was variable and remained low, there was no significant correlation with age.

**Figure 5:**
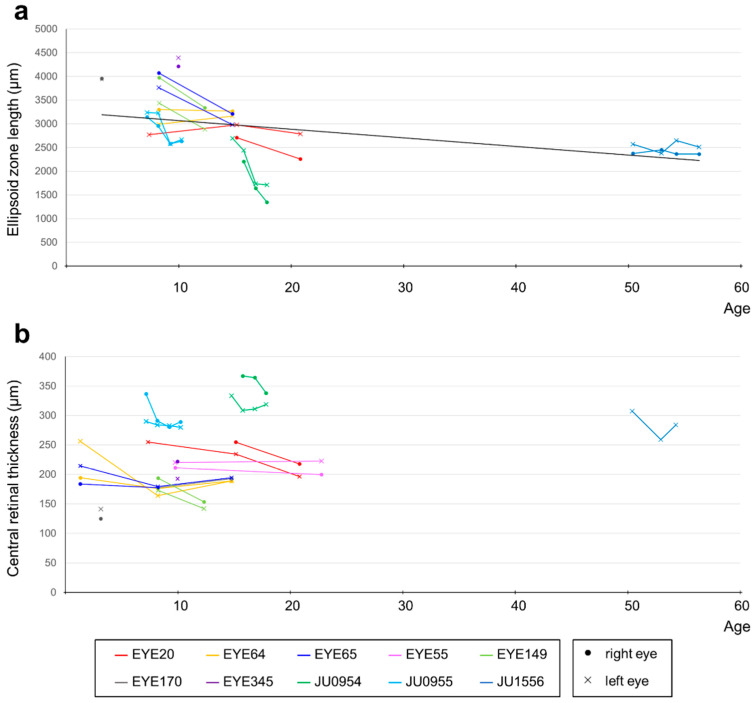
Correlation between OCT findings and age in patients with *RPGRIP1* variants. (**a**) The correlation between EZ length and age. Although EZ length showed a decreasing trend with age, it was maintained even after age 50. Different colors are used to represent each person, with dots indicating data for the right eye and crosses indicating data for the left eye. EZs that were maintained over the entire length of the imaging range were not plotted (EYE55 and parts of EYE64 and EYE65). (**b**) The correlation between central foveal retinal thickness and age. The right eye at all visits and the left eye at last visit of JU1556 were excluded because of abnormal thickening due to the epiretinal membrane. Although central retinal thickness varied from case to case, there seemed to be a trend of slow decrease with age in individual cases.

Decimal BCVA at the last visit varied from light perception to 0.2 (logMAR 0.7). The progression of the logMAR visual acuity in each case is shown in Figure 4. Although visual acuity improved a little in some cases until around age 12, visual acuity remained low and did not correlate with age. The visual field was unmeasurable in three cases, severely constricted at first examination in four cases (one of which progressed to unmeasurable), progressed from moderate to severe constriction in two cases, and mild constriction but relatively maintained in one case. Regarding ERG, all cases were non-recordable for the cone response at the first examination, but rod responses were reduced or subnormal in four cases and non-recordable in the remaining six cases. In the four cases in which the rod response remained, in three of the four cases the rod response disappeared on later re-examination, but in one case it remained. The retinal structure was evaluated for ellipsoid zone (EZ) length and central foveal retinal thickness. Figure 5a shows that the EZ length decreased with age (r = −0.728, *p* < 0.01) in 44 examinations of 18 eyes of nine patients, excluding one case with unimpaired EZ; however, it was maintained even in the case of the patient in their 50s. The central foveal retinal thickness of 19 eyes in 10 cases, excluding one eye with abnormal thickening due to traction by the epiretinal membrane, is shown in Figure 5b. Although the central retinal thickness varied from case to case, in individual cases, there seemed to be a trend of a slow decrease. The retinal structure was preserved over time relative to visual acuity in all cases.

**Figure 6:**
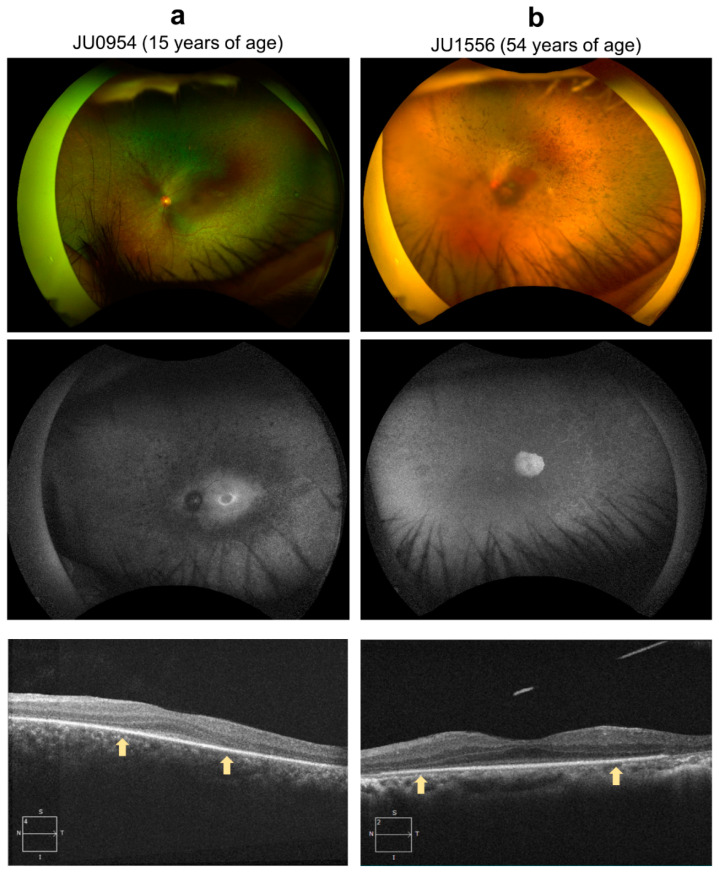
Color fundus photography, fundus autofluorescence (FAF), and optic coherence tomography (OCT). (**a**) The left eye of case JU0954, a 15-year-old female at the time of examination. Fundus examination showed narrowing of retinal vessels, diffuse retinal degeneration, and bone-spicule pigmentation; FAF showed a hyperfluorescent ring around the fovea, and hypofluorescence in the midperiphery where retinal degeneration was shown in fundus photograph; and OCT showed thinning of the outer nuclear layer and disruption of the peripheral EZ (outside of arrows). However, the central foveal retinal thickness was preserved. (**b**) The left eye of case JU1556, a 54-year-old female. Fundus examination shows almost complete obliteration of retinal vessels and extensive pigmentations with superior retinal dominance, which were consistent with a peripheral visual field defect; FAF showed hyperfluorescence in the macula area surrounded by multiple hypofluorescent spots from the arcade to periphery; and OCT showed thinning of the outer nuclear layer and disruption of the peripheral EZ (outside of arrows). However, central foveal retinal thickness was maintained.

Two representative examples are provided: Figure 6a shows the case of a 17-year-old female (JU0954). At nine months, the patient was brought to a nearby university hospital with a chief complaint of nystagmus and was diagnosed with LCA. At 14 years old, the patient visited Jikei University School of Medicine. The patient’s visual acuity remained low thereafter, and, at the patient’s last visit at age 17, her corrected visual acuity was hand-motion vision in the right eye and 0.01 in the left eye. Figure 6b shows the case of a 54-year-old female (JU1556). Six months after birth, the parents of the patient noticed no pursuit with her eyes and she was subsequently diagnosed with IRD at another university hospital. The patient was followed-up regularly until age 21. The patient had a maximum visual acuity of 0.07 from age 6 to 12 and 0.1 from age 13 to 21. In elementary school, the patient attended a school for blind students. The patient had no history of visiting an ophthalmologist between the ages of 21 and 45. Around the age of 45 years, the patient visited an ophthalmologist at a local doctor’s office with the chief complaint of progressive night blindness. At 50, the patient was referred to Jikei University School of Medicine for a detailed examination. At her last visit, visual acuity was 0.03 in her right eye and hand-motion vision in her left eye. Additionally, epiretinal membrane formation was observed in her right eye at the first visit and in the left eye at the last visit. In both cases, JU0954 and JU1556, ERG was absent in both cones and rods.

## 3. Discussion

This study successfully identified four copy number variations (CNVs) in four cases from three families using WGS (excluding the previously reported EYE55 with an exon 18 deletion). These CNVs involve only one to three exons. Therefore, detecting these CNVs using genome-wide CNV analysis of WES data may be difficult. To examine whether target CNV analysis for known disease-causing genes could detect these CNVs, we retrospectively examined CNV in *RPGRIP1* using jNord [27]. Microdeletions involving only exon 1 were not detected (JU0954, JU0955, and EYE170), as there were no baits on exon 1 (Figure 1b and Supplementary Figure S1). In contrast, a microdeletion involving exons 1–3 was detected as a deletion of exon 3 (JU1556). Microdeletions involving exon 18 were detected in two out of three cases. Therefore, these findings strongly support the idea that WES-based CNV analysis increases diagnostic yield; however, WGS is more reliable for CNV/SV analysis. A recent WES analysis of 1210 Japanese pedigree patients with inherited retinal diseases revealed no pathogenic variants of *RPGRIP1* [28]. CNVs involving *RPGRIP1* may be one of the genetic causes of IRD that were not detected by the WES-based analysis. There have been reports of the detection of non-coding variants such as CNVs and deep intron variants of *RPGRIP1* from cases unresolved via WES using WGS, indicating that non-coding variants may contribute significantly to *RPGRIP1*-related retinal dystrophy [7,9,11,15].

The causative genes of LCA, occurring at a frequency of approximately ≥10%, include *GUCY2D*, *RPE65*, *CRB1*, *CEP290*, and *RDH12* [3,4,5,29]. Previous reports have shown that the frequency of the causative genes varies according to the patient’s ethnic background [5,30]. Although *CEP290* is a common causative gene in patients of European ancestry, it was reported not to be as high in a large-scale study of patients of Chinese ancestry. In contrast, *RPGRIP1* is a more common causative gene in patients of Chinese ancestry than those of European ancestry [30,31,32]. In our previous study employing 34 Japanese families with LCA, most of the causative genes accounted for only one pedigree, except *CRB1* (three families), *NMNAT1* (three families), *RPGRIP1* (three families), and *GUCY2D* (two families) [19]. Although it is difficult to demonstrate the accurate frequency of *RPGRIP1* mutations in Japanese families with LCA because of the small number of cases and bias in the patient collection, we believe that *RPGRIP1* is possibly a common causative gene in Japanese patients with LCA.

Common pathogenic variants are crucial for genetic counseling of recessive diseases. A previous report showed that c.1107del (25 patients) and c. 2480G>T (12 patients) mutations were frequently isolated from patients with LCA6 [8]. The c.1107del mutation was prevalent in Saudi Arabia [33,34,35,36]. Consistent with a previous report, an exon 18 deletion in *RPGRIP1* was detected in six individuals from four families in Japan [19,25]. An exon 18 deletion was also identified in a recently published database of Japanese cohorts. Because the allele frequency is relatively high at 0.002%, it is considered a variant that should be noted in genetic counseling for LCA. Although examination by population, including Chinese and Koreans, is necessary, it is considered important for genetic counseling of patients of East Asian origin with LCA.

Gene-specific clinical trials using viral vectors are actively being conducted. In particular, the success of gene therapy for *RPE65* abnormalities is expected to be a breakthrough in this extremely serious disease [37,38,39]. Based on the experience of the long-term use of adeno-associated virus (AAV) vectors in Europe and the United States, a certain degree of safety has been ensured for the administration of viral vectors. It is a promising treatment due to its efficacy [40,41]. Gene therapy using AAV vectors in *RPGRIP1*-related murine and canine models has also been investigated [42,43]. Although LCA is a serious disease, it generally progresses slowly, and the patient’s visual function fluctuates daily, making it difficult to determine the therapeutic effect. The detailed clinical features were analyzed herein as basic data for future genetic therapy.

All participants had likely null variants such as splice site variants, nonsense variants, frameshift variants, and microdeletions; thus, we expected to show clinical trends. However, the results for visual function were variable. In our study, the visual field and refraction varied; visual acuity remained low and did not noticeably decline with age; and ERGs showed a loss of cone response and a decrease or loss of rod response in all cases.

The three siblings EYE20, 64, and 65 had marked nystagmus and photophobia from infancy and we diagnosed them with LCA. An ERG performed in the twin brothers at 15 months of age showed detectable rod responses [20]. However, we thought, since the twin brothers were under general anesthesia and at the young age of 1 year and 3 months, we could detect a significant response. When we re-examined them 13 years later, the rod responses were absent in all three patients. Biallelic variants of *RPGRIP1* are known to cause RP, CRD, and PPRCA in addition to LCA, and cases of isolated cone dysfunction were recently reported [44]. There have been reports of severe retinal degeneration with reduced but measurable rod response when examined at very young ages, as in our cases, suggesting that more severely affected cones than rods are characteristic of *RPGRIP1* [8,25].

Patient EYE55 first saw his previous doctor for marked nystagmus at age 7 months, but he was unable to undergo an ERG. He was followed up as LCA because his decimal BCVA did not increase beyond 0.1 until the age of 8 years. At age 9, ERG was performed under general anesthesia and the cone response was absent and rod response was decreased(subnormal) [20]. At age 20, the ERG was re-examined and the rod response remained. Other clinical findings were also better than in the other cases. Patient EYE55 was initially diagnosed with LCA, but long-term follow-up suggested a form of the disease similar to CRD.

The optical coherence tomography (OCT) findings of *RPGRIP1*-related retinal dystrophy confirmed that although EZ length and retinal thickness tended to decrease gradually, retinal structures were relatively preserved, and visual acuities were low but maintained, as in previous reports [8,10,13,20]. Interestingly, the retinal structure of the EZ was relatively preserved in a patient in her 50s with LCA. Based on an examination of the clinical picture, gene therapy, which greatly improves visual function in childhood, is promising.

A limitation of this study was the small number of cases, as LCA is an extremely rare disease. Regarding the examination of clinical features, there was a limit to the accuracy of ophthalmic examinations because most of the recruited patients were pediatric.

In summary, WGS is useful for detecting structural abnormalities and played a key role in the cohort examined in this study. Structural variants may be important genetic causes of *RPGRIP1*-related retinal dystrophy in Japanese patients. It is expected that the use of WGS will become mainstream in the future. Deletion of exon 18 is relatively common in the Japanese population, and genetic counseling for childhood IRDs requires careful attention.

## 4. Materials and Methods

### 4.1. Patient Recruitment

We performed WES on 29 participants from 28 families with clinically suspected LCA without systemic symptoms other than psychomotor delays. Among the 29 cases, 11 were newly recruited and 18 were unresolved cases from the previous target sequencing study. We identified causative variants in 12 cases using WES. We performed WGS for 14 unresolved cases. Among the sibling cases (JU0954 and 0955), we performed WGS in only one case (JU0955). The DNA samples in the remaining two cases were insufficient for WGS. Clinical data were collected from 10 Japanese patients with identified pathogenic variants of *RPGRIP1* who visited the National Center for Child Health and Development (NCCHD) and Jikei University School of Medicine.

### 4.2. WES and WGS

For WES, genomic DNA was captured using a SureSelect Human All Exon V6 kit (Agilent Technologies, Santa Clara, CA, USA) and sequenced on a NextSeq500 (Illumina, San Diego, CA, USA) with 151 bp paired-end reads. Data processing, variant calling, annotation, and filtering were performed as previously described [45]. Retrospective WES-based CNV analysis was performed using jNord [27]. WGS was commissioned by BGI Japan Corp. (Kobe, Japan). Sequencing was performed using DNBSEQ (MGI TECH, Shenzhen, China) with 150 bp paired-end reads. Data processing, variant calling, and variant annotation were performed as previously described [46]. CNV, SV, and transposon detection were performed using Canvas v1.40.0 [21], Manta v1.6.0 [22], and TEMP2 v0.1.4 [23], respectively. Alignments were visualized using the IGV v2.16.0 (downloaded from https://igv.org/). De novo assembly using aberrantly aligned read pairs, such as clipped reads, was performed using Megahit v1.2.9 [47]. The allele frequency of structural variation was examined using gnomAD SVs v2.1 (https://gnomad.broadinstitute.org/; accessed on 13 January 2023), 8.3KJPN-SV, based on short-read whole-genome sequencing of 8300 Japanese individuals, and JSV1, based on long-read whole-genome sequencing of 222 Japanese individuals (https://jmorp.megabank.tohoku.ac.jp/; accessed on 13 January 2023) [48].

### 4.3. Clinical Examination

The affected participants were examined at the NCCHD and Jikei University School of Medicine and underwent ophthalmic examinations, including BCVA, refraction measurement, kinetic visual fields on the Goldmann perimeter, and full-field ERG. Additionally, slit-lamp biomicroscopy, ophthalmoscopy after pupillary dilation, fundus photography, fundus autofluorescence (FAF) (Optos California, Optos Plc, Dunfermline, UK), and spectral-domain OCT (Cirrus HD-OCT 5000, Carl Zeiss Meditec AG, Dublin, CA, USA) or swept-source OCT (DRI OCT-1, Topcon Corporation, Tokyo, Japan) were performed. Information on family history was obtained through interviews with participants or their family members. BCVA was measured as decimal visual acuity using a Landolt C chart and converted to logMAR units for analysis. A picture chart was used for patients whose condition was difficult to examine using the Landolt C-chart. Visual acuity values for counting fingers, hand motion, and light perception were extrapolated to logMAR 2.0, 2.4, and 2.7, respectively [49]. The refractive error was recorded as a spherical equivalent and analyzed for each participant. BCVA and OCT were examined on multiple occasions in all affected subjects, except for OCT in cases EYE170 and 345, and phenotypes were analyzed by plotting BCVA versus age, central foveal retinal thickness versus age, and EZ length versus age. All statistical analyses were performed using SPSS version 25 (IBM Corp., Armonk, NY, USA). Spearman’s rank correlation coefficient was used for simple correlation tests. Statistical significance was set at *p* < 0.05.

## Figures and Tables

**Table 1:** List of patients with pathogenic variants of *RPGRIP1*(NM_020366.4).

Family ID	Patient ID	Identified Variant		Zygosity	Origin	Analysis Method	Reference
EYE20	EYE20	c.3565_3571del, p.(Arg1189Glyfs*7)		hom	biparental	TS	[24]
	EYE64	c.3565_3571del, p.(Arg1189Glyfs*7)		hom	biparental	TS	[24]
	EYE65	c.3565_3571del, p.(Arg1189Glyfs*7)		hom	biparental	TS	[24]
EYE55	EYE55	c.1467+1G>T		het	paternal	TS	[20]
		c.2710+374_2895+78del	(ex 18 del)	het	maternal	PCR	[25]
EYE149	EYE149	c.799C>T, p.(Arg267*)		het	paternal	TS	[26]
		c.1687C>T, p.(Arg563*)		het	maternal		[19]
EYE170	EYE170	c.3565_3571del, p.(Arg1189Glyfs*7)		het	paternal	WES, WGS	[24]
		g.21276834_21281300del	(ex 1 del)	het	maternal		Novel
EYE345	EYE345	c.871_872ins[N[(300_400)]; 854_871]		het	paternal	WES, WGS	Novel
		c.1363del, p.(Glu455Lysfs*2)		het	maternal		Novel
JIKEI-122	JU0954	c.2710+374_2895+78del	(ex 18 del)	het	paternal	WES	[25]
		g.21276147_21280265del	(ex 1 del)	het	maternal		Novel
	JU0955	c.2710+374_2895+78del	(ex 18 del)	het	paternal	WES, WGS	[25]
		g.21276147_21280265del	(ex 1 del)	het	maternal		Novel
JIKEI-1556	JU1556	c.2710+374_2895+78del	(ex 18 del)	het	paternal	WES, WGS	[25]
		g.21277271_21297204delinsN [330]	(ex 1–3 del)	het	maternal		Novel

ex: exon; del: deletion; hom: homozygous; het: heterozygous; TS: Targeted sequencing; PCR: polymerase chain reaction; WES: Whole-exome sequencing; WGS: Whole-genome sequencing.

**Table 2:** Summary of ophthalmological measurements of patients with *RPGRIP1* variants.

Patient	Family ID		EYE20			EYE55	EYE149	EYE170	EYE345	JIKEI-122		JIKEI-1556
	Patient ID		EYE20	EYE64	EYE65	EYE55	EYE149	EYE170	EYE345	JU0954	JU0955	JU1556
	Gender		F	M	M	M	M	F	M	F	M	F
Decimal BCVA	at the first test	Age	2	3	3	2	7	3	9	3	3	50
		OD	0.04	0.03	0.04	0.03	HM	LP	HM	0.01	0.05	0.04
		OS	0.03	0.02	0.04	0.04	HM	LP	HM	HM	0.05	0.02
	at the last test	Age	20	14	14	22	18	10	13	17	10	56
		OD	0.08	0.05	0.06	0.2	0.01	LP	LP	HM	0.1	0.03
		OS	0.09	0.06	0.06	0.2	0.01	LP	LP	0.01	0.09	HM
Refractive error (D)		OD	+5.25	+5.00	−2.00	−5.75	+5.00	-	-	+7.00	+1.75	+2.00
(spherical equivalent)		OS	+7.00	+4.00	−2.85	−5.00	+5.00	-	-	+7.00	+1.75	+1.25
Electroretinogram		Age	7	1	1	9	8	3	2	8	2	50
		Rod	reduced	subnormal	subnormal	subnormal	ND	ND	ND	ND	ND	ND
		Cone	ND	ND	ND	ND	ND	ND	ND	ND	ND	ND
	re-examination	Age	20	14	14	22						
		Rod	ND	ND	ND	subnormal						
		Cone	ND	ND	ND	ND						
Visual Field (V4e)		Age	20	14	14	22				17	10	55
		OD	5°	5°	5°	65°	-	-	-	ND	7°	5°
		OS	5°	8°	5°	70°	-	-	-	ND	7°	3°
OCT		Age	20	14	14	22	12	3	9	17	10	56
	EZ length (μm)	OD	2255	3268	3208	intact	3338	3953	4210	1346	2632	2362
		OS	2788	3160	2982	intact	2882	3940	4390	1711	2667	2510
	CRT (μm)	OD	218	189	193	199	153	125	222	338	289	*
		OS	196	189	194	223	142	141	192	318	279	284

Visual field and OCT are listed as of the last examination. BCVA: best-corrected visual acuity; OD: oculus dexter; OS: oculus sinister; LP: light perception; HM: hand motion; ND: not detectable; OCT: optical coherence tomography; EZ: ellipsoid zone; CRT: central retinal thickness. * Excluded from analysis because of abnormal thickening due to epiretinal membrane.

## Data Availability

The data presented in this study are available on request from the corresponding author.

## Supplementary Materials

The following supporting information can be downloaded at: https://www.mdpi.com/article/10.3390/ijms241813678/s1.

## References

[1] Koenekoop R.K. (2004). An overview of Leber congenital amaurosis: A model to understand human retinal development. Surv. Ophthalmol..

[2] Schappert-Kimmijser J., Henkes H.E., Van Den Bosch J. (1959). Amaurosis congenita (Leber). AMA Arch. Ophthalmol..

[3] den Hollander A.I., Roepman R., Koenekoop R.K., Cremers F.P. (2008). Leber congenital amaurosis: Genes, proteins and disease mechanisms. Prog. Retin. Eye Res..

[4] Kumaran N., Moore A.T., Weleber R.G., Michaelides M. (2017). Leber congenital amaurosis/early-onset severe retinal dystrophy: Clinical features, molecular genetics and therapeutic interventions. Br. J. Ophthalmol..

[5] Huang C.H., Yang C.M., Yang C.H., Hou Y.C., Chen T.C. (2021). Leber’s Congenital Amaurosis: Current Concepts of Genotype-Phenotype Correlations. Genes.

[6] Dryja T.P., Adams S.M., Grimsby J.L., McGee T.L., Hong D.H., Li T., Andréasson S., Berson E.L. (2001). Null RPGRIP1 alleles in patients with Leber congenital amaurosis. Am. J. Hum. Genet..

[7] Jamshidi F., Place E.M., Mehrotra S., Navarro-Gomez D., Maher M., Branham K.E., Valkanas E., Cherry T.J., Lek M., MacArthur D. (2019). Contribution of noncoding pathogenic variants to RPGRIP1-mediated inherited retinal degeneration. Genet. Med..

[8] Beryozkin A., Aweidah H., Carrero Valenzuela R.D., Berman M., Iguzquiza O., Cremers F.P.M., Khan M.I., Swaroop A., Amer R., Khateb S. (2021). Retinal Degeneration Associated with RPGRIP1: A Review of Natural History, Mutation Spectrum, and Genotype-Phenotype Correlation in 228 Patients. Front. Cell Dev. Biol..

[9] Zou G., Zhang T., Cheng X., Igelman A.D., Wang J., Qian X., Fu S., Wang K., Koenekoop R.K., Fishman G.A. (2021). Noncoding mutation in RPGRIP1 contributes to inherited retinal degenerations. Mol. Vis..

[10] Mao Y., Long Y., Liu B., Cao Q., Li Y., Li S., Wang G., Meng X., Li S. (2021). Ocular Characteristics of Patients with Leber Congenital Amaurosis 6 Caused by Pathogenic RPGRIP1 Gene Variation in a Chinese Cohort. J. Ophthalmol..

[11] Perrault I., Hanein S., Gerard X., Mounguengue N., Bouyakoub R., Zarhrate M., Fourrage C., Jabot-Hanin F., Bocquet B., Meunier I. (2021). Whole Locus Sequencing Identifies a Prevalent Founder Deep Intronic RPGRIP1 Pathologic Variant in the French Leber Congenital Amaurosis Cohort. Genes.

[12] Liu Z., Wang H., He X., Tao D., Li L. (2023). Identifying two pathogenic variants in a patient with pigmented paravenous retinochoroidal atrophy. Open Life Sci..

[13] Li T. (2014). Leber congenital amaurosis caused by mutations in RPGRIP1. Cold Spring Harb. Perspect. Med..

[14] Won J., Gifford E., Smith R.S., Yi H., Ferreira P.A., Hicks W.L., Li T., Naggert J.K., Nishina P.M. (2009). RPGRIP1 is essential for normal rod photoreceptor outer segment elaboration and morphogenesis. Hum. Mol. Genet..

[15] Fadaie Z., Whelan L., Ben-Yosef T., Dockery A., Corradi Z., Gilissen C., Haer-Wigman L., Corominas J., Astuti G.D.N., de Rooij L. (2021). Whole genome sequencing and in vitro splice assays reveal genetic causes for inherited retinal diseases. NPJ Genom. Med..

[16] Carss K.J., Arno G., Erwood M., Stephens J., Sanchis-Juan A., Hull S., Megy K., Grozeva D., Dewhurst E., Malka S. (2017). Comprehensive Rare Variant Analysis via Whole-Genome Sequencing to Determine the Molecular Pathology of Inherited Retinal Disease. Am. J. Hum. Genet..

[17] Hussain H.M.J., Wang M., Huang A., Schmidt R., Qian X., Yang P., Marra M., Li Y., Pennesi M.E., Chen R. (2023). Novel Pathogenic Mutations Identified from Whole-Genome Sequencing in Unsolved Cases of Patients Affected with Inherited Retinal Diseases. Genes.

[18] Wen S., Wang M., Qian X., Li Y., Wang K., Choi J., Pennesi M.E., Yang P., Marra M., Koenekoop R.K. (2023). Systematic assessment of the contribution of structural variants to inherited retinal diseases. Hum. Mol. Genet..

[19] Hosono K., Nishina S., Yokoi T., Katagiri S., Saitsu H., Kurata K., Miyamichi D., Hikoya A., Mizobuchi K., Nakano T. (2018). Molecular Diagnosis of 34 Japanese Families with Leber Congenital Amaurosis Using Targeted Next Generation Sequencing. Sci. Rep..

[20] Miyamichi D., Nishina S., Hosono K., Yokoi T., Kurata K., Sato M., Hotta Y., Azuma N. (2019). Retinal structure in Leber’s congenital amaurosis caused by RPGRIP1 mutations. Hum. Genome Var..

[21] Roller E., Ivakhno S., Lee S., Royce T., Tanner S. (2016). Canvas: Versatile and scalable detection of copy number variants. Bioinformatics.

[22] Chen X., Schulz-Trieglaff O., Shaw R., Barnes B., Schlesinger F., Källberg M., Cox A.J., Kruglyak S., Saunders C.T. (2016). Manta: Rapid detection of structural variants and indels for germline and cancer sequencing applications. Bioinformatics.

[23] Yu T., Huang X., Dou S., Tang X., Luo S., Theurkauf W.E., Lu J., Weng Z. (2021). A benchmark and an algorithm for detecting germline transposon insertions and measuring de novo transposon insertion frequencies. Nucleic Acids Res..

[24] Seong M.W., Kim S.Y., Yu Y.S., Hwang J.M., Kim J.Y., Park S.S. (2008). Molecular characterization of Leber congenital amaurosis in Koreans. Mol. Vis..

[25] Suzuki T., Fujimaki T., Yanagawa A., Arai E., Fujiki K., Wada Y., Murakami A. (2014). A novel exon 17 deletion mutation of RPGRIP1 gene in two siblings with Leber congenital amaurosis. Jpn. J. Ophthalmol..

[26] Li L., Xiao X., Li S., Jia X., Wang P., Guo X., Jiao X., Zhang Q., Hejtmancik J.F. (2011). Detection of variants in 15 genes in 87 unrelated Chinese patients with Leber congenital amaurosis. PLoS ONE.

[27] Uchiyama Y., Yamaguchi D., Iwama K., Miyatake S., Hamanaka K., Tsuchida N., Aoi H., Azuma Y., Itai T., Saida K. (2021). Efficient detection of copy-number variations using exome data: Batch- and sex-based analyses. Hum. Mutat..

[28] Suga A., Yoshitake K., Minematsu N., Tsunoda K., Fujinami K., Miyake Y., Kuniyoshi K., Hayashi T., Mizobuchi K., Ueno S. (2022). Genetic characterization of 1210 Japanese pedigrees with inherited retinal diseases by whole-exome sequencing. Hum. Mutat..

[29] Sweeney M.O., McGee T.L., Berson E.L., Dryja T.P. (2007). Low prevalence of lecithin retinol acyltransferase mutations in patients with Leber congenital amaurosis and autosomal recessive retinitis pigmentosa. Mol. Vis..

[30] Wang H., Wang X., Zou X., Xu S., Li H., Soens Z.T., Wang K., Li Y., Dong F., Chen R. (2015). Comprehensive Molecular Diagnosis of a Large Chinese Leber Congenital Amaurosis Cohort. Investig. Ophthalmol. Vis. Sci..

[31] Coppieters F., Casteels I., Meire F., De Jaegere S., Hooghe S., van Regemorter N., Van Esch H., Matuleviciene A., Nunes L., Meersschaut V. (2010). Genetic screening of LCA in Belgium: Predominance of CEP290 and identification of potential modifier alleles in AHI1 of CEP290-related phenotypes. Hum. Mutat..

[32] Astuti G.D., Bertelsen M., Preising M.N., Ajmal M., Lorenz B., Faradz S.M., Qamar R., Collin R.W., Rosenberg T., Cremers F.P. (2016). Comprehensive genotyping reveals RPE65 as the most frequently mutated gene in Leber congenital amaurosis in Denmark. Eur. J. Hum. Genet..

[33] Khan A.O., Abu-Safieh L., Eisenberger T., Bolz H.J., Alkuraya F.S. (2013). The RPGRIP1-related retinal phenotype in children. Br. J. Ophthalmol..

[34] Khan A.O., Al-Mesfer S., Al-Turkmani S., Bergmann C., Bolz H.J. (2014). Genetic analysis of strictly defined Leber congenital amaurosis with (and without) neurodevelopmental delay. Br. J. Ophthalmol..

[35] Eisenberger T., Neuhaus C., Khan A.O., Decker C., Preising M.N., Friedburg C., Bieg A., Gliem M., Charbel Issa P., Holz F.G. (2013). Increasing the yield in targeted next-generation sequencing by implicating CNV analysis, non-coding exons and the overall variant load: The example of retinal dystrophies. PLoS ONE.

[36] Abu-Safieh L., Alrashed M., Anazi S., Alkuraya H., Khan A.O., Al-Owain M., Al-Zahrani J., Al-Abdi L., Hashem M., Al-Tarimi S. (2013). Autozygome-guided exome sequencing in retinal dystrophy patients reveals pathogenetic mutations and novel candidate disease genes. Genome Res..

[37] Weleber R.G., Pennesi M.E., Wilson D.J., Kaushal S., Erker L.R., Jensen L., McBride M.T., Flotte T.R., Humphries M., Calcedo R. (2016). Results at 2 Years after Gene Therapy for RPE65-Deficient Leber Congenital Amaurosis and Severe Early-Childhood-Onset Retinal Dystrophy. Ophthalmology.

[38] Bainbridge J.W., Mehat M.S., Sundaram V., Robbie S.J., Barker S.E., Ripamonti C., Georgiadis A., Mowat F.M., Beattie S.G., Gardner P.J. (2015). Long-term effect of gene therapy on Leber’s congenital amaurosis. N. Engl. J. Med..

[39] Lorenz B., Tavares J., van den Born L.I., Marques J.P., Pilotto E., Stingl K., Charbel Issa P., Leroux D., Dollfus H., Scholl H.P.N. (2023). Current Management of Inherited Retinal Degeneration Patients in Europe: Results of a 2-Year Follow-Up Multinational Survey by the European Vision Institute Clinical Research Network—EVICR.net. Ophthalmic Res..

[40] Timmers A.M., Newmark J.A., Turunen H.T., Farivar T., Liu J., Song C., Ye G.J., Pennock S., Gaskin C., Knop D.R. (2020). Ocular Inflammatory Response to Intravitreal Injection of Adeno-Associated Virus Vector: Relative Contribution of Genome and Capsid. Hum. Gene Ther..

[41] Bucher K., Rodríguez-Bocanegra E., Dauletbekov D., Fischer M.D. (2021). Immune responses to retinal gene therapy using adeno-associated viral vectors—Implications for treatment success and safety. Prog. Retin. Eye Res..

[42] Pawlyk B.S., Bulgakov O.V., Liu X., Xu X., Adamian M., Sun X., Khani S.C., Berson E.L., Sandberg M.A., Li T. (2010). Replacement gene therapy with a human RPGRIP1 sequence slows photoreceptor degeneration in a murine model of Leber congenital amaurosis. Hum. Gene Ther..

[43] Lhériteau E., Petit L., Weber M., Le Meur G., Deschamps J.Y., Libeau L., Mendes-Madeira A., Guihal C., François A., Guyon R. (2014). Successful gene therapy in the RPGRIP1-deficient dog: A large model of cone-rod dystrophy. Mol. Ther..

[44] Khan A.O. (2023). RPGRIP1-related retinal disease presenting as isolated cone dysfunction. Ophthalmic Genet..

[45] Tachibana N., Hosono K., Nomura S., Arai S., Torii K., Kurata K., Sato M., Shimakawa S., Azuma N., Ogata T. (2022). Maternal Uniparental Isodisomy of Chromosome 4 and 8 in Patients with Retinal Dystrophy: SRD5A3-Congenital Disorders of Glycosylation and RP1-Related Retinitis Pigmentosa. Genes.

[46] Hiraide T., Shimizu K., Miyamoto S., Aoto K., Nakashima M., Yamaguchi T., Kosho T., Ogata T., Saitsu H. (2022). Genome sequencing and RNA sequencing of urinary cells reveal an intronic FBN1 variant causing aberrant splicing. J. Hum. Genet..

[47] Li D., Liu C.M., Luo R., Sadakane K., Lam T.W. (2015). MEGAHIT: An ultra-fast single-node solution for large and complex metagenomics assembly via succinct de Bruijn graph. Bioinformatics.

[48] Tadaka S., Hishinuma E., Komaki S., Motoike I.N., Kawashima J., Saigusa D., Inoue J., Takayama J., Okamura Y., Aoki Y. (2021). jMorp updates in 2020: Large enhancement of multi-omics data resources on the general Japanese population. Nucleic Acids Res..

[49] Lange C., Feltgen N., Junker B., Schulze-Bonsel K., Bach M. (2009). Resolving the clinical acuity categories “hand motion” and “counting fingers” using the Freiburg Visual Acuity Test (FrACT). Graefes Arch. Clin. Exp. Ophthalmol..

