# Supplementary material for: The Structural Abnormalities Are Deeply Involved in the Cause of RPGRIP1-Related Retinal Dystrophy in Japanese Patients
Source: Int J Mol Sci. 2023 Sep 5;24(18):13678. doi: 10.3390/ijms241813678 (PMC10531429; doi:10.3390/ijms241813678)

Supplementary Figure S1. CNV analysis of *RPGRIP1* by jNord

CNV calls are highlighted in red color.

EYE170: c.3565_3571del + <u>Exon 1 deletion</u>

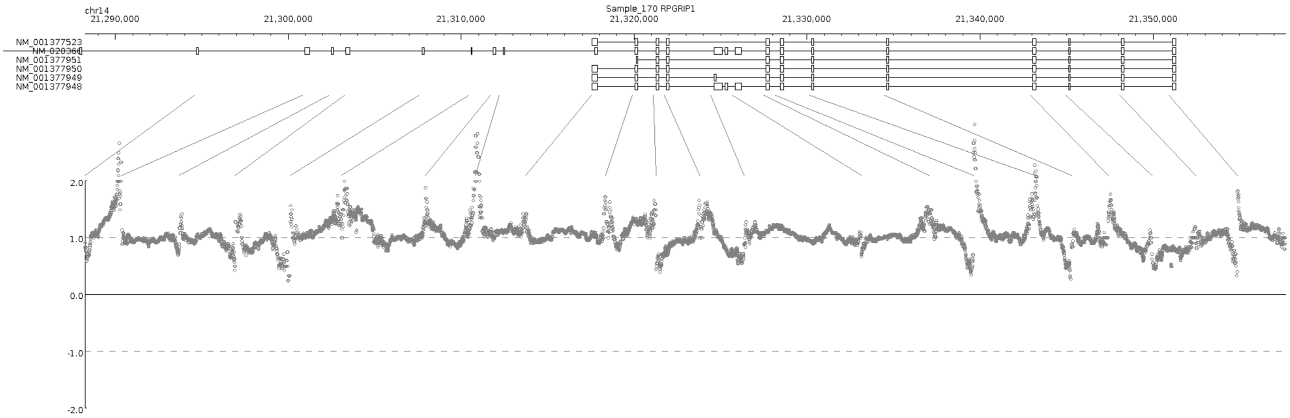

EYE345: c.1363del + *Alu* insertion (No CNV)

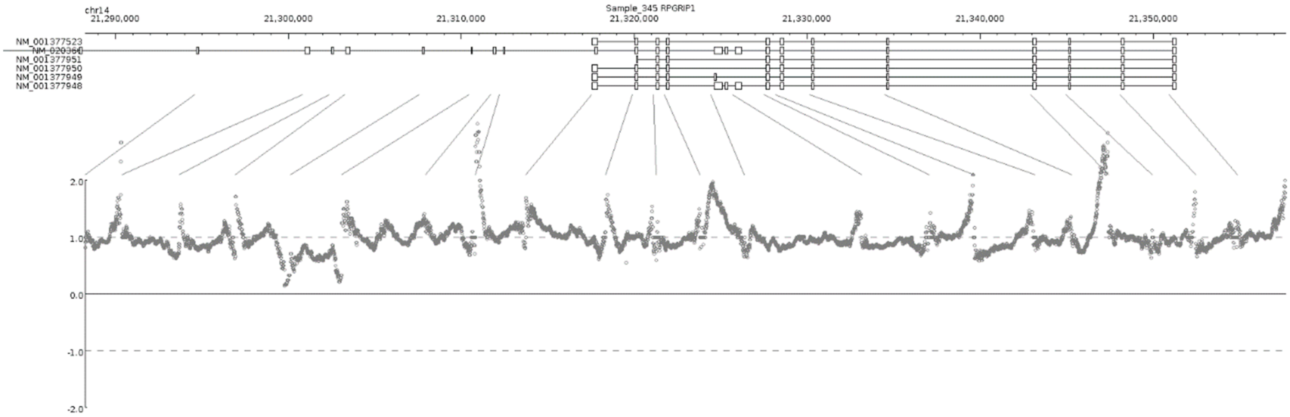

JU1556: <u>Exon 1-3 deletion</u> + <u>Exon 18 deletion</u>

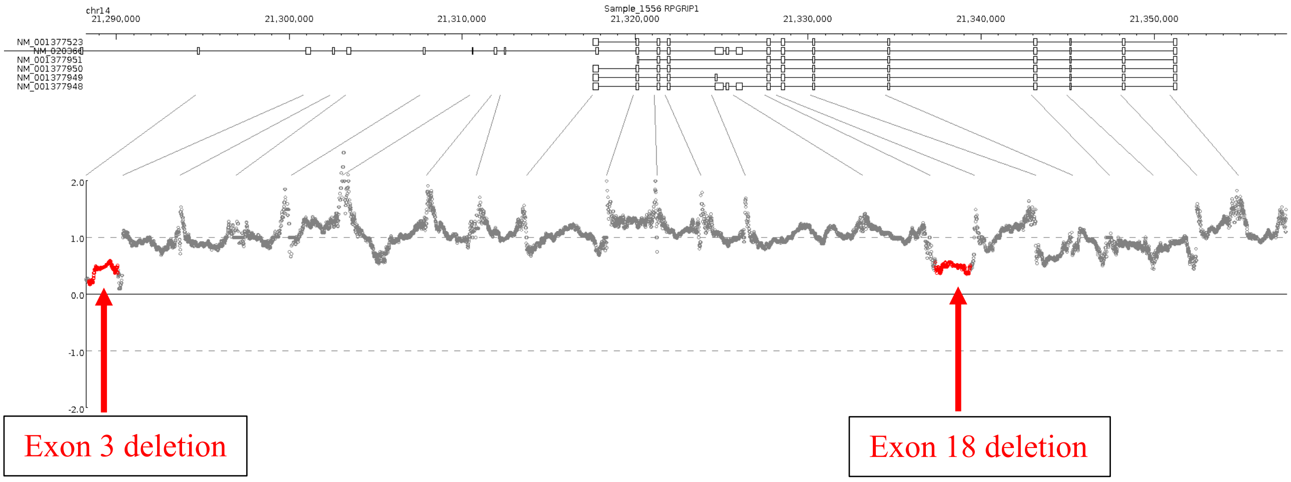

JU954: <u>Exon 1 deletion</u> + <u>Exon 18 deletion</u>

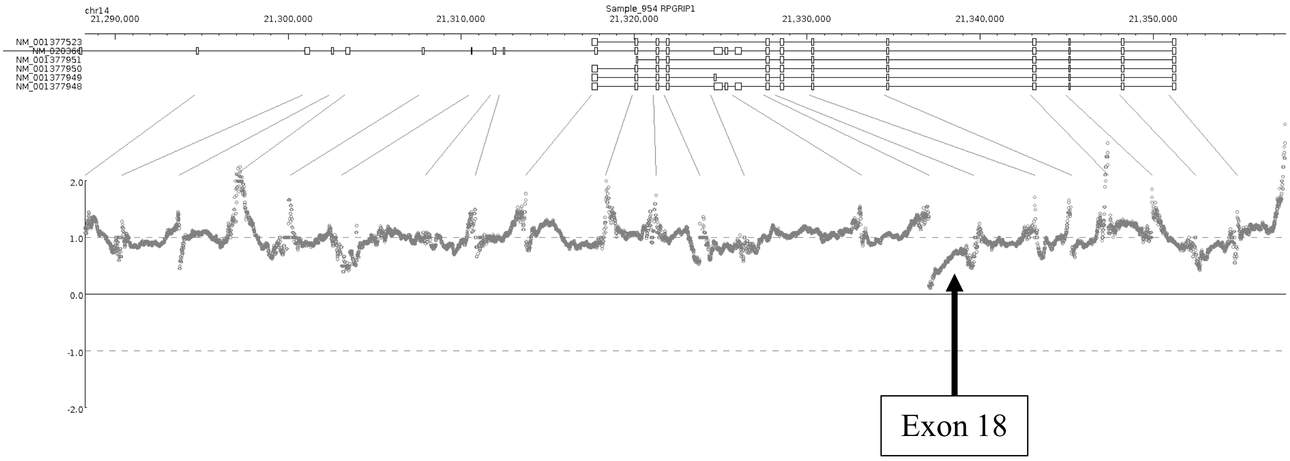

JU955: <u>Exon 1 deletion</u> + <u>Exon 18 deletion</u>

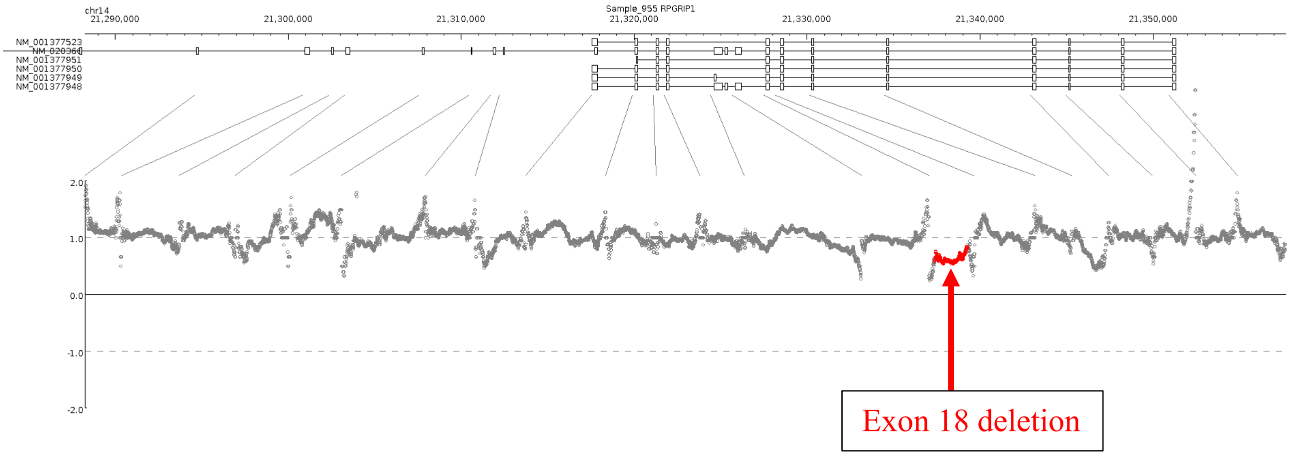

Supplement: Supplementary file 1 [file ijms-24-13678-s001.zip › Supp_Figure_S1.pdf]
